# RAPSO: An Integrated PSO with Reinforcement Learning and an Adaptive Weight Strategy for the High-Precision Milling of Elastic Materials

**DOI:** 10.3390/s25185913

**Published:** 2025-09-22

**Authors:** Qingxin Li, Peng Zeng, Qiankun Wu, Zijing Zhang

**Affiliations:** 1 State Key Laboratory of Robotics, Shenyang Institute of Automation, Chinese Academy of Sciences, Shenyang 110169, China; zp@sia.cn (P.Z.); wuqiankun@sia.cn (Q.W.); zhangzijing@sia.cn (Z.Z.); 2 University of Chinese Academy of Sciences, Beijing 101408, China

**Keywords:** robot path planning, particle swarm optimization, milling of elastic materials

## Abstract

This study tackles the challenge of achieving high-precision robotic machining of elastic materials, where elastic recovery and overcutting often impair accuracy. To address this, a novel milling strategy, RAPSO, is introduced by combining an adaptive particle swarm optimization (APSO) algorithm with a reinforcement learning (RL)-based compensation mechanism. The method builds a material-specific milling model through residual error characterization, incorporates a dynamic inertia weight adjustment strategy into APSO for optimized toolpath generation, and integrates a Proximal Policy Optimization (PPO)-based RL module to refine trajectories iteratively. Experiments show that RAPSO reduces residual material by 33.51% compared with standard PSO and APSO methods, while offering faster convergence and greater stability. The proposed framework provides a practical solution for precision machining of elastic materials, offering improved accuracy, reduced post-processing requirements, and higher efficiency, while also contributing to the theoretical modeling of elastic recovery and advanced toolpath planning.

## 1. Introduction

High-precision robotic machining of elastic materials (e.g., viscoelastic polymers, composite propellants, and flexible rubbers) has become a critical requirement in advanced manufacturing fields such as aerospace, biomedical engineering, and soft robotics. However, the inherent deformability and post-machining elastic recovery of these materials, coupled with the flexible dynamic characteristics of robotic systems, lead to persistent challenges including dimensional deviations, overcutting/undercutting, and trajectory instability—all of which severely impair machining accuracy and surface integrity [1,2]. Cetin et al. (2024) further verified the effectiveness of machine learning algorithms in predicting material wear, providing algorithmic references for elastic material machining error prediction [3]. To address these issues, the integration of swarm intelligence optimization, machine learning, and adaptive control has emerged as a promising direction, though existing research still leaves key gaps in practical applicability.

### 1.1. State of the Art in Elastic and Flexible Material Machining

The machining of deformable materials has long relied on modeling and optimization to mitigate accuracy losses. Early foundational work by Bravo et al. (2005) established a landmark model for milling stability that simultaneously considers the flexibility of both the workpiece and the machine tool, revealing how dynamic interactions between the two contribute to vibration-induced errors [4]. Budak (2006) built an analytical model for cutting forces and structural deformations, laying a basic framework for subsequent elastic material machining modeling [5]. Building on this, Campa et al. (2011) focused on thin-wall floor milling with bull-nose end mills, proposing a chatter avoidance framework that combines geometric modeling and stability diagrams to reduce deformation-related defects [6]. Their findings highlighted the need for material-specific process adaptation, a theme expanded by Marin (2024) in five-axis milling of free-form surfaces; his research demonstrated that tool geometry (ball-end vs. circle-segment end mills) and material flexibility jointly determine surface quality, though it lacked real-time adjustment mechanisms for elastic recovery [7].

In terms of process parameter optimization, Varga et al. (2024) quantified the influence of tool inclination angle and effective cutting speed on the roughness of machined flexible surfaces, providing empirical guidance for parameter selection but not addressing trajectory-level optimization [8]. For a more systematic process simulation, Urbikain-Pelayo et al. (2025) developed Mill+, a tool integrating vibration, cutting force, and surface quality prediction—yet its static modeling approach fails to capture the dynamic springback of elastic materials during robotic machining [9]. These studies collectively confirm that while flexibility and deformation are well-recognized, existing models often decouple material behavior from robotic system dynamics, limiting their precision in practical scenarios.

### 1.2. Path Planning and Optimization for Machining

Swarm intelligence algorithms, particularly particle swarm optimization (PSO), have been widely adopted for machining path optimization due to their efficiency in global searches. Zhang et al. (2020) proposed a PSO-RBFNN hybrid algorithm for industrial robot inverse kinematics, improving motion smoothness but relying on fixed parameters that struggle with elastic material variability [10]. Bai et al. (2025) optimized the structural stability of CNC gantry machine tools through multi-objective optimization, indirectly providing a hardware foundation for path planning accuracy improvement [11]. To enhance PSO’s performance, fusion with other meta-heuristics has been explored: Zhao et al. (2024) applied an SA-PSO (simulated annealing-PSO) algorithm to optimize impeller side milling paths, boosting efficiency by 15% but failing to balance early-stage exploration and late-stage exploitation [12]. Li et al. (2021) combined PSO with LSSVM for surface roughness prediction, expanding PSO’s application in machining quality control [13]. Abualigah (2025) summarized the latest advances in PSO, noting that adaptive parameter adjustment is key to improving its optimization performance [14]. Pajaziti et al. (2025) optimized CNC machine paths and performance via PSO, further verifying PSO’s role in improving machining efficiency [15].

Adaptive strategies have emerged as a solution to fixed-parameter limitations. Abbas et al. (2020) developed an adaptive design for stainless steel face milling that balances cost, quality, and productivity, while Kaood et al. (2021) optimized nanofluid performance via adaptive PSO—yet neither addressed the unique springback characteristics of elastic materials [16,17]. Even advanced path planning methods, such as the jerk-limited heuristic feedrate scheduling proposed by Xiao et al. (2022), prioritize trajectory smoothness over dynamic error compensation for deformable workpieces [18]. Liu et al. (2024) proposed a linear programming-based time-optimal feedrate planning method, and Liu et al. (2025) developed an S-shaped feedrate-based NURBS interpolator, further enriching the technical path of feedrate optimization [19,20]. El-Kenawy et al. (2022) and Bashir et al. (2023) validated the effectiveness of ensemble learning in engineering applications of intelligent optimization algorithms, providing algorithmic references for machining parameter optimization [21,22].

### 1.3. Reinforcement Learning and Dynamic Compensation

Reinforcement learning (RL) has shown promise for real-time control in complex machining scenarios, thanks to its ability to learn from environmental feedback. Xiao et al. (2019) was among the first to apply meta-reinforcement learning to machining parameter optimization, laying a foundation for subsequent research [23]. Lu et al. (2025) used meta-RL for energy-efficient five-axis flank milling, reducing energy consumption by 20% while maintaining precision; however, their approach was not tailored to elastic material springback [2]. Zhang et al. (2024) applied deep RL to machining process route planning, improving robustness but lacking a mechanism for trajectory-level error correction [24].

Kaliyannan et al. (2024) combined RL with deep learning for tool condition monitoring, providing technical support for real-time diagnosis of machining processes [25]. Danket et al. (2025) used deep RL to achieve adaptive production capacity planning under variable electricity costs, demonstrating RL’s adaptability in dynamic production environments [26]. Dynamic error compensation remains a critical bottleneck. While Mantas et al. (2025) integrated AI with digital twins for carbon fiber-reinforced plastics (CFRP) machining, their system relied on pre-calibrated models rather than real-time adaptive compensation [27]. For robotic systems specifically, Makulavičius et al. (2023) noted that robotic flexibility—while enabling complex operations—introduces spasmodic trajectory movements during continuous interpolation, a problem rarely addressed in existing compensation frameworks [28].

### 1.4. Critical Gaps in Existing Research

Despite significant progress, three core limitations persist in the high-precision milling of elastic materials:

- Inadequate Springback Modeling. Most existing models (e.g., [6,9]) rely on simplified assumptions or empirical coefficients for elastic recovery, failing to capture its dynamic dependence on cutting thickness and material properties. For example, Liu et al. (2004) established a micro-end-milling force model but did not extend it to predict post-machining springback in viscoelastic materials [29].
- Unbalanced Optimization Performance.Traditional PSO and even adaptive variants (e.g., [12,17]) use static or semi-static inertia weights, leading to either premature convergence (local optima) or slow convergence—which are critical issues for time-sensitive robotic machining.
- Lack of Robot-Material Adaptive Compensation. While RL-based methods (e.g., [2,24]) improve path robustness, they do not integrate real-time feedback of elastic recovery and robotic trajectory fluctuations, resulting in persistent overcutting/undercutting.

### 1.5. Objectives and Contributions of This Study

The main contributions of this article can be summarized as follows:

- Residual Definition Milling Model. A resilient milling model based on residual definition is constructed, which can predict the deformation and rebound behavior of elastic materials. This model provides a theoretical foundation for addressing machining accuracy issues caused by material characteristics during the milling process of elastic materials, contributing to improved dimensional accuracy and surface quality of the final product.
- Adaptive Weight PSO Algorithm. An improved particle swarm optimization algorithm is proposed, which incorporates an adaptive inertia weight strategy for path planning. By dynamically adjusting the inertia weight, this algorithm can broadly explore the solution space in the early stages and refine the search later on, accelerating convergence and improving cutting precision.
- Compensation Module Based on Reinforcement Learning. A reinforcement learning compensation module using Proximal Policy Optimization (PPO) is integrated. This module is capable of dynamically adjusting strategies based on real-time feedback, reducing processing errors caused by springback and overcutting, thereby further enhancing machining accuracy and surface smoothness.

This paper is structured as follows: Section 2 presents the theoretical foundation, detailing the development of an elastic material machining model grounded in residual error analysis alongside the formulation of process constraints and optimization criteria. Subsequently, Section 3 introduces a hybrid particle swarm optimization (PSO) framework integrating reinforcement learning principles and adaptive inertia weight mechanisms, named as RAPSO, to generate high-precision cutting trajectories. Furthermore, Section 4 validates the proposed methodology through comparative simulations across diverse machining scenarios, quantitatively assessing its performance in trajectory generation efficiency and dimensional precision. Finally, Section 5 summarizes the key findings and implications of this research, highlighting the technical contributions and potential applications in precision manufacturing.

## 2. Identifying Dynamic Parameters

A material-specific deformation–prediction framework is established to characterize the elastic recovery and structural deformation dynamics of workpieces during precision machining. This model integrates a quantitative metric for uncut material volume, which serves as the primary optimization criterion for the subsequent reinforcement learning-based trajectory refinement process.

### 2.1. Material-Specific Machining Behavior Analysis

**Figure 1:**
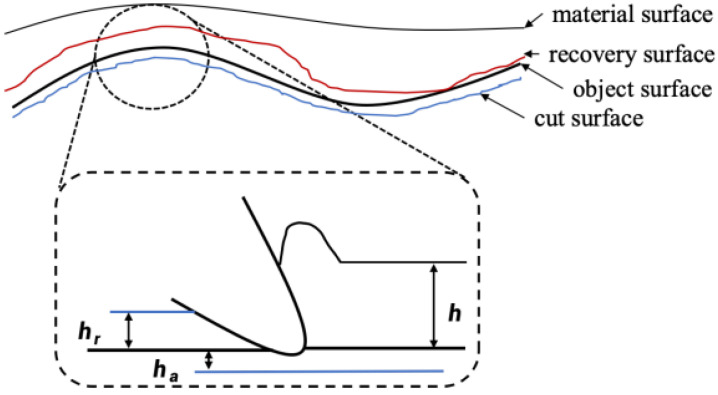
Rebound phenomenon in milling elastic materials.

As illustrated in the schematic diagram (Figure 1), workpieces composed of viscoelastic materials exhibit distinctive machining behavior due to their inherent stress relaxation properties. During the contouring operation towards the target geometry [29], the material undergoes post-machining elastic recovery, resulting in dimensional deviations from the desired profile. By incorporating this springback phenomenon into the toolpath planning algorithm (represented by the optimized trajectory in the diagram), the final surface contour achieves improved conformity with the design specifications through compensatory adjustments.

**Figure 2:**
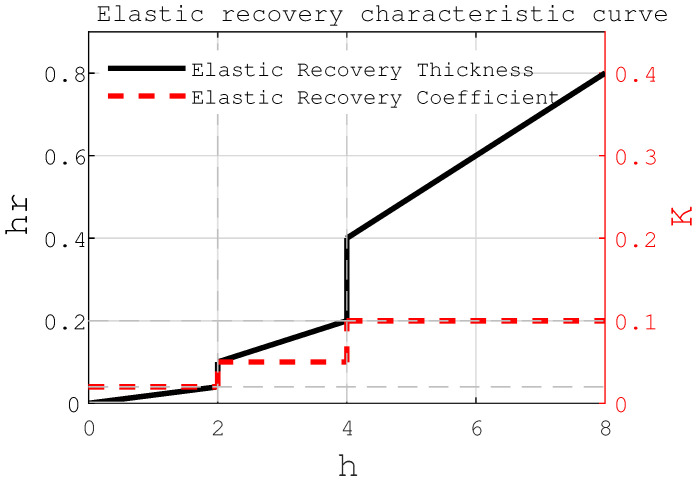
Relationship diagram between elastic recovery amount, elastic recovery coefficient, and cutting thickness.

To account for elastic recovery during machining, a thickness-dependent elastic recovery coefficient *k* is introduced:

(1) $$\begin{array}{cc} \; & \frac{h_{r}}{h}=k\\ \; & \left\{\;\begin{array}{cc} k=0.1, & h>h_{l}\\ k=0.02, & h<h_{min}\\ k=0.05, & h_{min}<h<h_{l} \end{array}\right. \end{array}$$

where $h_{r}$ denotes the thickness recovered elastically after the cutting process, *h* represents the nominal cutting thickness, and *k* is the coefficient characterizing the elastic recovery behavior. Based on the principle of minimum cutting thickness, the value of k is not constant but varies with the actual cutting thickness. In the present study, *k* is defined as a function of *h*, following the expression given above, with the reference thickness $h_{l}=4$ mm and the minimum effective cutting thickness $h_{min}=2$ mm. This functional dependence allows the model to account for size effects and material behavior at small undeformed chip thicknesses. Although empirically defined, this approach is grounded in machining principles such as the minimum cutting thickness and viscoelastic material behavior [29]. By incorporating *k* as a function of *h*, the model more accurately predicts post-machining deviations. The relationship between elastic recovery, *k*, and cutting thickness is illustrated in Figure 2, supporting both theoretical analysis and experimental validation.

### 2.2. Milling Allowance Definition

**Figure 3:**
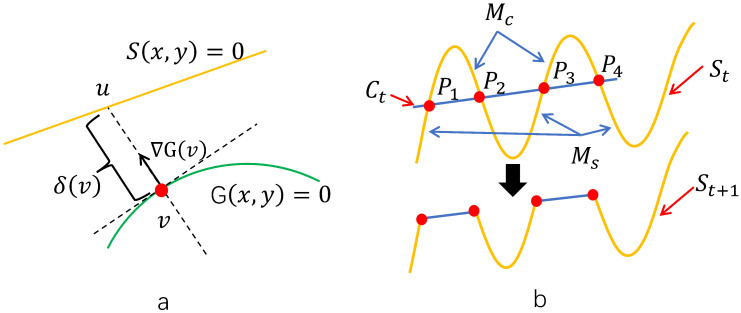
Description of cutting process. (**a**) Definition of milling allowance; (**b**) The process of material edge shape change during cutting.

In the machining of elastic materials, the term “milling allowance” refers to the predetermined layer of material intentionally left for removal to compensate for elastic deformations induced during the milling operation. This allowance is critical for achieving the desired dimensional precision and surface integrity in the final component. Materials exhibiting significant elasticity, such as specific types of rubber or compliant polymers, are prone to undergo reversible deformation when subjected to cutting forces. This phenomenon, illustrated in Figure 3, necessitates careful planning of the machining allowance to ensure that the elastic recovery is adequately accommodated within the process parameters.

In precision machining processes, the desired geometry of the workpiece is typically defined using CAD models. However, such representations, whether in the form of boundary meshes or discrete point clouds, are often unsuitable for direct integration into mathematical and physical simulations of the cutting process. To facilitate analytical treatment, a continuous implicit function representation is adopted. The target geometry is thereby described by the zero level set of a scalar function:

(2) G(x,y)=0

The actual initial geometry of the workpiece is represented by another implicit function:

(3) S(x,y)=0

In the context of cutting path optimization, the material boundary plays a pivotal role in influencing both the process efficiency and geometric fidelity. This boundary is defined as the planar curve separating the region to be retained from the excess material designated for removal. It can be accurately reconstructed through piecewise functional fitting techniques based on sampled edge data.

A key objective in process planning is to generate a toolpath that minimizes material waste while maintaining high cutting precision. Such a toolpath is initially specified as a sequence of discrete waypoints that the cutting tool must traverse. To ensure smooth and continuous motion, these discrete points are interpolated into a continuous curve that closely conforms to the actual material edge.

As illustrated in Figure 3, the gradient vector of the target shape function G(x,y), denoted as follows:

(4) $$▽G=\left(G_{x},G_{y}\right)=\left(\frac{\partial G}{\partial x},\frac{\partial G}{\partial y}\right)$$

Equation (4) defines the local normal direction at any point v on the target curve. The corresponding point u on the initial workpiece surface S(x,y)=0 along this normal direction satisfies the geometric relation, as follows:

(5) $$\vec{u}=\vec{v}+\alpha ▽G\left(v\right)$$

Given that *u* lies on the surface *S*, it follows that S(u)=0. Substituting Equation (5) into the implicit form of *S* yields the following:

(6) $$S\left(u\right)=S(\vec{v}+\alpha ▽G\left(v\right))=0$$

For a given point *v*, Equation (6) can be solved for the scalar parameter α. Defining α=f(v), the local thickness of the material to be removed at position *v* is subsequently determined as follows:

(7) $$\delta^{\left(s\right)}{\left(v\right)}_{v\in X^{\left(G\right)}}=\left|\right|\vec{u}-\vec{v}\left|\right|=\alpha ||▽G\left(v\right)||=f\left(v\right)||▽G\left(v\right)||$$

### 2.3. Cutting Constraints and Objectives

Machining operations are inherently irreversible material-removal processes, where any excess material removed beyond the designated boundary cannot be restored. To preserve the integrity of the final component geometry, it is essential to prevent overcutting, which occurs when the tool path intersects or penetrates the target shape boundary. Consequently, a fundamental process constraint requires that the cutting tool trajectory must remain entirely outside or coincident with the desired final surface but never intrude into the region bounded by the target curve. Therefore, the cutting constraint can be defined as follows:

(8) $$\delta^{\left(s\right)}\left(v\right)\geq 0,\forall v\in X^{\left(G\right)}$$

Furthermore, the primary objective of the machining operation is to efficiently eliminate the surplus material defined by the initial workpiece geometry, thereby approaching the final target shape as closely as possible. This entails minimizing the volume or area of residual material remaining after cutting, which directly correlates with machining accuracy and process efficiency. Accordingly, the optimization goal is formulated in Equation (9) to reduce the total material deviation between the initial stock and the desired contour, ensuring high precision and minimal post-processing requirements.

(9) $$\begin{array}{cc} \; & argmin:t\\ \; & \left\{\begin{array}{c} \max_{v\in X^{\left(G\right)}}\delta^{\left(S_{t}\right)}\left(v\right)\leq \epsilon \\ \min_{v\in X^{\left(G\right)}}\delta^{\left(S_{t}\right)}\left(v\right)\geq 0 \end{array}\right. \end{array}$$

Under the prescribed cutting accuracy tolerance ϵ and subject to the constraint defined by Equation (8), the optimization objective is to minimize the total number of cutting tools, denoted as t, required to complete the machining process. To improve efficiency while keeping precision within bounds.

## 3. Methodology

To address the machining characteristics of elastic materials, this study proposes a cutting trajectory planning method based on an improved particle swarm optimization algorithm. Owing to the elastic recovery of materials after the removal of cutting forces, direct machining according to the theoretical contour would result in deviations between the actual formed dimensions and the design specifications. To resolve this issue, the present work implements active compensation of tool paths through the optimization algorithm, introducing a dynamic adjustment mechanism into the conventional particle swarm optimization framework, thereby effectively mitigating precision loss caused by springback effects.

### 3.1. Particle Swarm Optimization Algorithm

For trajectory planning in robotic milling operations, this paper proposes a path optimization method based on feature point extraction. Specifically, the discrete tool center positions are first interpolated using spline curves to construct continuously differentiable machining trajectories. Subsequently, an adaptive sampling algorithm is employed to identify critical feature points along the path, which significantly reduces trajectory data volume while maintaining machining accuracy. This study adopts a segmented optimization strategy for milling trajectory planning, with the specific implementation process used being the following: First, based on machining accuracy requirements, the target curve S(x,y)=0 is discretized into *N* characteristic segments, and N+1 representative control nodes (including start/end points and key intermediate points) are selected to construct the initial trajectory. Within the optimization algorithm framework, these control nodes are mapped to particle swarm individuals in a high-dimensional search space, where each particle’s position is denoted as $P_{ix}=\left(1,2,\ldots ,N+1\right)$, velocity as $v_{i}$, and historical optimal position as $P_{best_{i}}$. Notably, as shown in Equation (7), the machining allowance δ serves as a key optimization index, whose magnitude exhibits a negative correlation with final surface quality. By utilizing a cutting simulation surrogate model, we can obtain the remaining material allowance after each cutting process. The fitness function for the particle swarm is defined as follows:

(10) $$f=\sum_{i=1}^{N+1}\delta^{\left(s_{t}\right)}\left(P_{i}\right)$$

Using the aforementioned equation, the minimum fitness value for each particle is determined, and the corresponding optimal trajectory position sequence, denoted as $P_{iy}$, can be derived. Consequently, the algorithmic formulation for the milling path in the particle swarm optimization is established.

(11) $$\left\{\begin{array}{c} v_{i}^{m+1}=wv_{i}^{m}+c_{1}r_{1}\left(P_{best_{i}}-P_{iy}^{m}\right)+c_{2}r_{2}\left(G_{best_{i}}-P_{iy}^{m}\right)\\ P_{iy}^{m+1}=P_{iy}^{m}+P_{iy}^{m} \end{array}\right.$$

where *w* denotes the inertia weight, reflecting the extent of reliance on the current velocity direction. The constants $c_{1}$ and $c_{2}$ are acceleration coefficients that control the maximum step size during the learning process. Additionally, $r_{1}$ and $r_{2}$ represent two independently generated random numbers uniformly distributed within [0, 1], introduced to enhance the stochastic nature of the search behavior.

### 3.2. Adaptive Weight Particle Swarm Optimization Algorithm

In the conventional particle swarm optimization (PSO) algorithm, the inertia weight remains constant and does not vary with the number of iterations. When the inertia weight is set to a large value during the initial stages, although it accelerates the convergence speed, it also increases the likelihood of the algorithm getting trapped in a local optimal solution, thereby hindering its ability to reach the global optimum. To address this issue, this paper introduces an adaptive inertia weight strategy that is adjusted based on the optimization progress, as shown in Equation (12).

(12) $$w=w_{min}+\left(1-\frac{i}{m}\frac{P_{best_{i}}}{G_{best_{i}}}\right)\left(w_{max}-w_{min}\right)$$

Given the parameters $w_{min}=0.6$ and $w_{max}=0.8$, where *m* denotes the total number of iterations and *i* represents the current iteration count, the ratio $\frac{i}{m}$ progressively increases as the algorithm executes, indicating that the computational process advances as expected. In the dynamic regulation mechanism of particle swarm optimization algorithms, parameter $\frac{P_{best_{i}}}{G_{best_{i}}}$ characterizes the proximity between the current particle fitness and the global optimal solution, with its dynamic variation reflecting the adaptive adjustment characteristics of the algorithm’s search strategy. Specifically, when the particle fitness approaches the global optimum, the inertia weight is reduced to contract the search range, enabling a precise local search within the neighborhood of the optimal solution. Conversely, during the initial algorithm phase, a larger inertia weight preserves the particle’s global exploration capability, ensuring comprehensive traversal of the solution space. This parameter regulation mechanism, based on variations in the value of $\frac{P_{best_{i}}}{G_{best_{i}}}$ and coupled with the relationship of $\frac{P_{best_{i}}}{G_{best_{i}}}$ and $1-\frac{i}{m}*\frac{P_{best_{i}}}{G_{best_{i}}}$, dynamically balances the requirements of global exploration and local exploitation. It achieves an adaptive transition from coarse-grained search to refined optimization, fully demonstrating the self-organizing characteristics of intelligent optimization algorithms.

### 3.3. Reinforcement Learning Optimization Module

To address the springback phenomenon and overcutting issues that occur during the milling of elastic materials, based on the aforementioned APSO algorithm, this paper further proposes an optimization method combined with reinforcement learning. Generally speaking, the initial cutting path is obtained based on APSO. For the particles (i.e., the path) in APSO, further compensation is carried out based on reinforcement learning to form the final compensated path.

(13) $$min_{x}L_{total}=L_{PSO}\left(x\right)+\lambda L_{PPO}\left(x\right)$$

**Figure 4:**
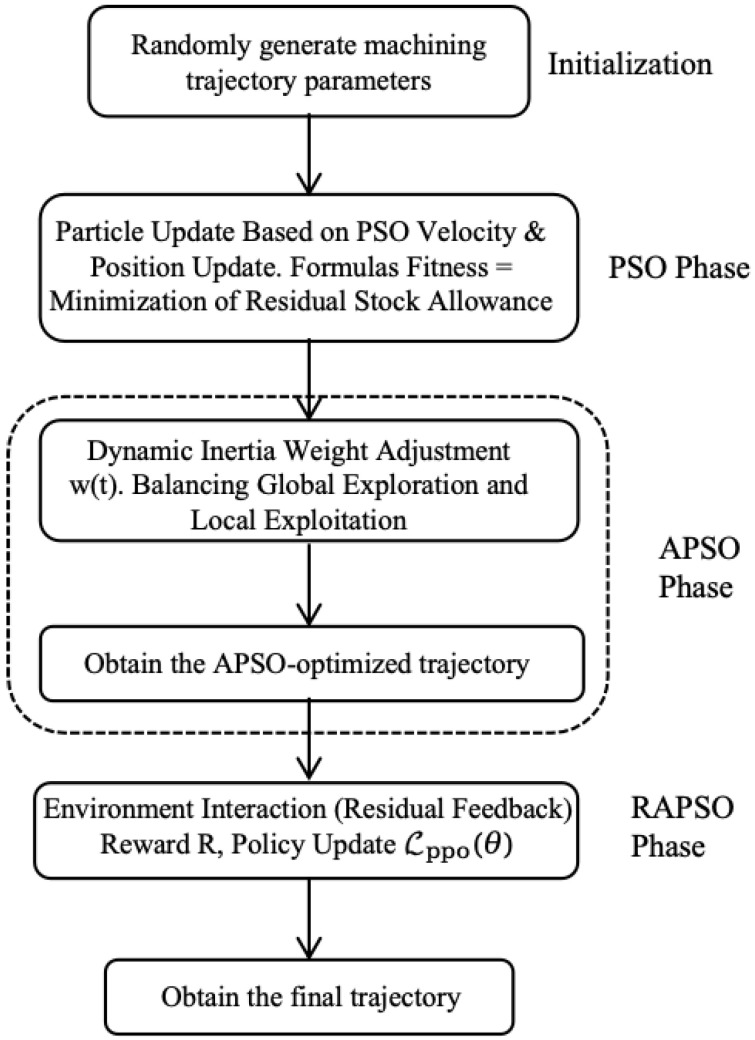
Optimized overall flowchart.

In this study, the Proximal Policy Optimization (PPO) algorithm, illustrated in Figure 4, was adopted to train our reinforcement learning model. The PPO algorithm is widely applied in various tasks due to its stability and efficiency. We simulate the milling process as an environment, input the position of each particle as the state to the model, and use the compensation amount output by the model to adjust the original path to reduce processing errors.

The reward function is the core of the reinforcement learning algorithm, determining the optimization direction of the compensation module. We design a multi-objective reward function that simultaneously optimizes processing precision, surface quality, and processing efficiency:

(14) $$R_{t}=-\left(\delta_{t}^{+}+\delta_{t}^{-}\right)-\lambda \cdot \Pi \left\{flag_{t}=0\right\}$$

where $\delta_{t}^{+}$ and $\delta_{t}^{-}$ are computed by evaluating the deviation of the recovered boundary from the ideal cutting edge. λ is a fixed penalty (set to 5.0) for invalid cutting (i.e., incomplete or failed operations). $\Pi \{flag_{t}=0\}$ is an indicator function, which takes a value of 1 when the $flag_{t}$ indicates a machining failure state, and 0 otherwise.

This formulation implicitly enforces a policy that prefers actions resulting in minimal recovery error and successful cuts, guiding the agent toward fine-tuned compensation distributions that preserve geometric fidelity post-cut. Although the current implementation uses fixed scalar weights λ, the reward function structure is inherently multi-objective:

(15) $$R=w_{1}\cdot \Delta_{reduction}-w_{2}\cdot \delta_{overcut}-w_{3}\cdot \delta_{uncercut}-w_{4}\cdot \mathrm{FailurePenaltv}$$

The physical meanings and functions of each indicator are as follows:

(1) Residual Reduction ($\Delta_{reduction}$): Represents the reduction in cutting residuals before and after compensation, serving as the core performance metric. A larger value indicates a more significant decrease in residual errors after compensation, with the optimization objective being maximization. In the reward function, it acts as a positive term, reinforced by the weight $w_{1}$ to incentivize improved accuracy.

(2) Overcut Amount ($\delta_{overcut}$): Quantifies the extent to which the actual trajectory exceeds the target boundary, measured in pixel units. Overcutting leads to unintended material removal and is considered a negative indicator. In the reward function, it is penalized through the negative weight $w_{2}$ to drive the optimization process toward minimizing this value.

(3) Undercut Amount ($\delta_{undercut}$): Quantifies the shortfall distance between the actual toolpath and the target boundary, measured in millimeter/pixel units. Undercutting results in residual material remaining on the workpiece surface after machining. Acts as a negative term penalized by weight $w_{3}$. The optimization objective is to minimize to ensure first-pass machining success.

(4) Machining Failure Penalty (FailurePenalty):A fixed-magnitude penalty is triggered when critical failures occur. It delivers a substantial negative reward upon failure detection. Moreover, it takes precedence over other objectives (safety-critical).

The PPO algorithm is adopted to train the policy network through the following steps and the complete execution flow is detailed in Algorithm 1:

(1) Environment Interaction:The agent interacts with the milling environment, executing action $a_{t}$ and observing immediate reward $r_{t}$ and new state $s_{t+1}$.

(2) Experience Replay:Store state–action–reward–state (SAR) transitions in an experience buffer for subsequent training.

(3) Objective Function Optimization:Use the PPO clip objective function for policy updates, limiting the difference between new and old policies to ensure training stability:

(16) $$L_{PPO}\left(\theta \right)=E\left[min\left(r_{t}\right)\left(\theta \right){\hat{A}}_{t},clip\left(r_{t}\left(\theta \right),1-\epsilon ,1+\epsilon \right)A_{t}\right]$$

where θ represents the current policy parameters. $r_{t}\left(\theta \right)=\pi_{\theta }\left(a_{t}|s_{t}\right)/\pi_{\theta_{\mathrm{old}}}\left(a_{t}|s_{t}\right)$ is the probability ratio between the new and old policies. ${\hat{A}}_{t}$ is the estimated advantage function, typically computed using Generalized Advantage Estimation (GAE). ϵ is a small threshold (e.g., 0.2) controlling the clipping range.

This formulation encourages the agent to improve the policy based on the advantage estimation while constraining large deviations from the previous policy, ensuring training stability.

The PPO loss is typically composed of three parts:

(17) $$L=L_{clip}-c_{1}\cdot L_{value}+c_{2}\cdot L_{entropy}$$

(4) Value Network Update:Use Generalized Advantage Estimation (GAE) to estimate the advantage function and update the Critic network.

(5) Dynamic Weight Adjustment:Based on historical reward values and processing goals, dynamically adjust the weights of the multi-objective reward function to achieve adaptive optimization.
**Algorithm 1** PPO-based Milling Path Compensation Module1:**Input:** Prepared tool path image set $S=\{s_{0},\;s_{1},\ldots ,s_{n}\}$. Initial policy parameters $\theta_{0}$, value network parameters $\phi_{0}$. Action set $A=\{a_{1},\;a_{2},\ldots ,a_{k}\}$. Maximum iterations *T*, clipping coefficient ϵ, learning rate α.2:**Output:** Trained policy $\pi_{\theta }$ for generating compensation sequence.3:Initialize policy network $\pi_{\theta }$ and value network $V_{\phi }$ with $\theta_{0},\;\phi_{0}$.4:**for**t=1 to *T* **do**5:  Sample image $s_{t}\in S$ and initialize $E_{t}$6:  **for** each time step in episode **do**7:     Select action $a_{t}∼\pi_{\theta }\left(a_{t}|s_{t}\right)$8:     Execute $a_{t}$ in $E_{t}$, observe reward $r_{t}$ and $s_{t+1}$9:     Store transition $\left(s_{t},\;a_{t},\;r_{t},\;s_{t+1}\right)$ into buffer *B*10:   **end for**11:   Compute advantage estimates ${\hat{A}}_{t}$ using GAE12:   Compute policy ratio $r_{t}\left(\theta \right)=\pi_{\theta }\left(a_{t}|s_{t}\right)/\pi_{\theta_{\mathrm{old}}}\left(a_{t}|s_{t}\right)$13:   Optimize clipped PPO objective14:   Update policy network parameters θ using gradient ascent15:   Update value network parameters ϕ via MSE loss16:**end for**17:**return** optimized policy $\pi_{\theta }$

## 4. Experiment and Analysis

### 4.1. Typical Scenarios

**Figure 5:**
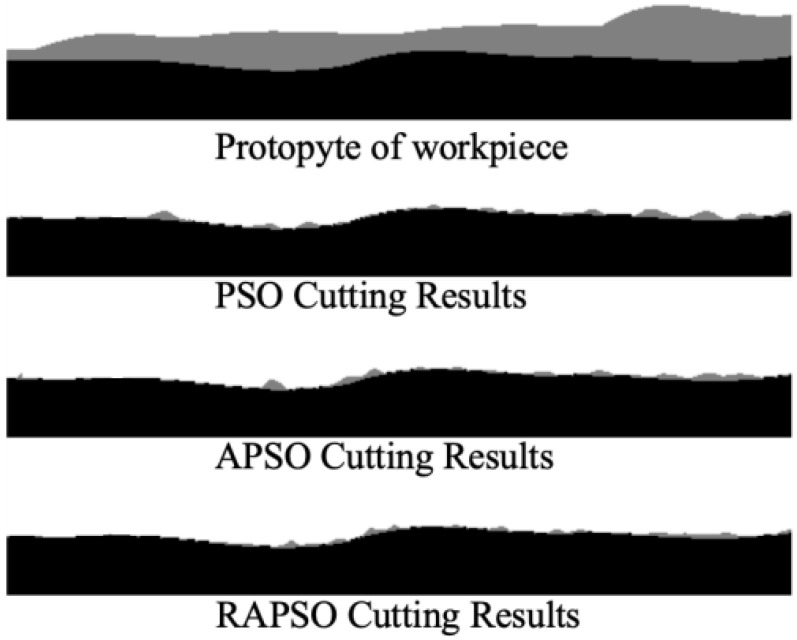
Comparison of milling processing results.

Taking the workpiece in Figure 5 as an example, the black part represents the target shape to be machined, and the gray part represents the part to be cut. The optimal cutting path obtained through the traditional particle swarm optimization algorithm retains a greater margin than the RAPSO after cutting.

As shown in Figure 5, the comparison of processing results among PSO, APSO (Adaptive PSO), and RAPSO clearly presents the differences in path optimization effects. The processing trajectory generated by RAPSO is closer to the target boundary, and the material residual after cutting is the smallest.

**Figure 6:**
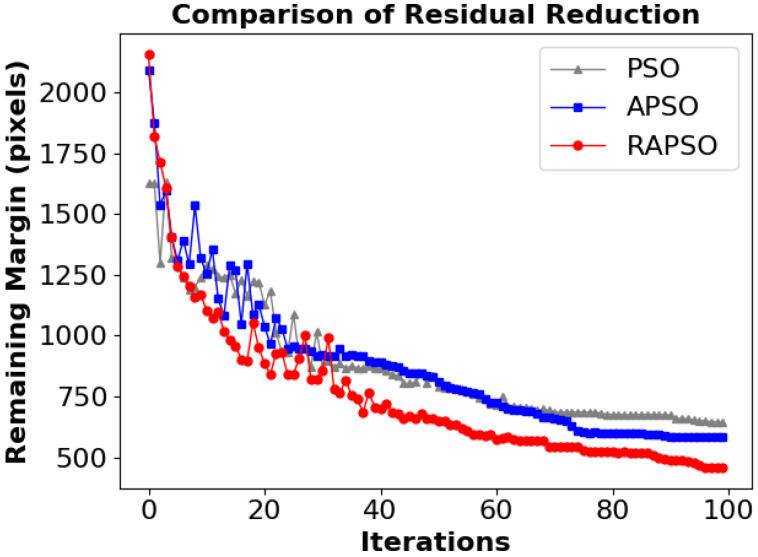
Remaining change.

Furthermore, as shown in Figure 6, during 100 iterations, the variation trends of residual allowances corresponding to the three algorithms with the number of iterations are presented. The convergence speed of RAPSO is significantly higher than that of PSO and APSO, and it shows obvious advantages in the early stage, which reflects the effectiveness of the reinforcement learning compensation strategy.

### 4.2. Multiple Scene Statistics Results

**Figure 7:**
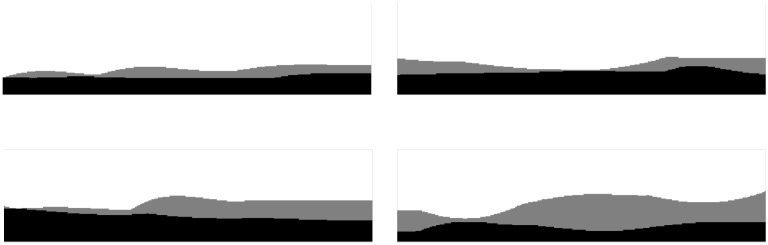
Randomly generated four processing cases.

To further verify the universality of the algorithm in processing tasks of different complex workpieces, this paper randomly selects four typical machining cases (Figure 7) for comparative experiments and records the residual allowances (unit: pixel) of PSO, APSO, and RAPSO under their respective optimal solutions. The experimental results are shown in Table 1.

According to Table 1, the incorporation of the adaptive weight adjustment strategy enables APSO to reduce residual pixels by an average of 6.50% compared to the traditional PSO algorithm. On this basis, RAPSO further integrates the reinforcement learning path compensation mechanism, which can reduce the error by an average of 28.47%, thus significantly improving the accuracy and stability of path planning. It can be concluded that the improved algorithm has enhanced cutting accuracy.

### 4.3. Experimental Validation of Robotic Machining System

**Figure 8:**
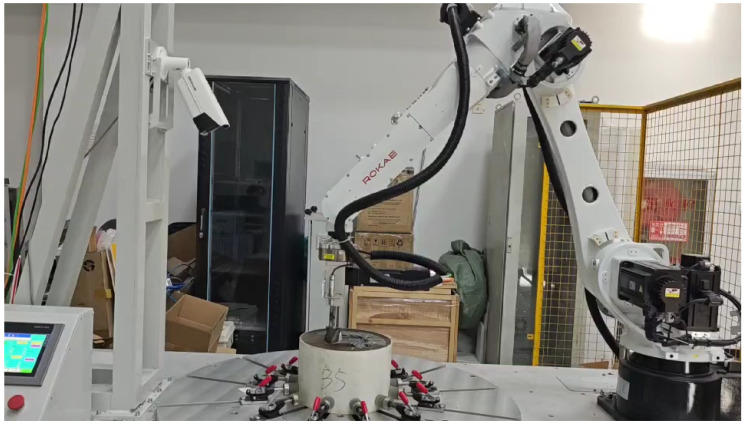
Robot milling experimental platform.

**Figure 9:**
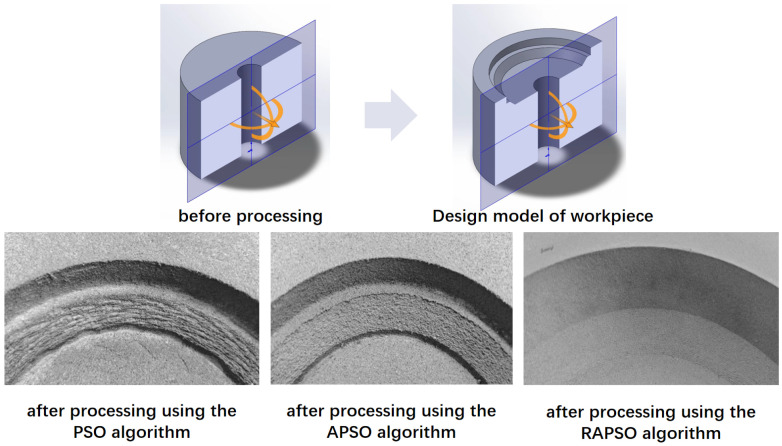
Comparison of milling experiments.

To empirically validate the proposed methodologies, a comprehensive robotic machining system was developed, illustrated in Figure 8, comprising a high-precision six-axis manipulator (Rokae NB25, Rokae Robotics Co., Ltd., Beijing, China, repeatability ±0.03 mm, payload 25 kg), a 3D depth-sensing camera array (Mech-Mind, Mech-Mind Robotics Co., Ltd., Beijing, China spatial resolution 0.05 mm, field of view 500×500 mm), a trajectory generation workstation, and a CNC-end milling unit (maximum spindle speed 20,000 rpm, 2.2 kW). A double-edged ball milling cutter was used with a rotation speed of 100 r/s and a feed speed of 0.5 mm/s. The 3D imaging subsystem acquires detailed surface topography through point clouds, which are cross-referenced with the target STL model to identify discrete machining layers for processing. The trajectory generation workstation implements the two proposed algorithms to synthesize optimized toolpaths, coordinating robotic arm movements and spindle parameters in real time. Experimental results (Figure 9) demonstrate that all methods achieve dimensional accuracy within the specified tolerance (≤2 mm). These algorithms effectively mitigate two critical challenges in elastic material machining: localized overcutting during tool engagement and residual deformation (springback) during unloading. The improved surface finish further confirms the effectiveness of the algorithms in maintaining dimensional stability and preserving geometric integrity throughout iterative machining cycles.

## 5. Conclusions

This study introduces RAPSO, a hybrid optimization framework for high-precision robotic milling of elastic materials, which integrates adaptive weight particle swarm optimization (PSO) with a PPO-based reinforcement learning module. By incorporating a residual-based milling model, dynamic inertia weight adjustment, and learning-based trajectory compensation, RAPSO effectively addresses challenges such as springback and overcutting. Experimental results demonstrate that RAPSO reduces residual material by 33.51% compared with standard PSO, achieves faster convergence, and maintains higher stability, thereby improving machining accuracy and efficiency and reducing post-processing requirements.

The proposed framework provides both practical and theoretical contributions. It offers a robust solution for the precision machining of elastic materials while advancing the modeling of elastic recovery and enabling more effective toolpath planning. Future work will focus on further refining elastic recovery and milling allowance modeling through empirical formulas combined with multi-fidelity Kriging surrogate models and evaluating RAPSO in more diverse industrial environments, including different materials, tools, and multi-axis milling scenarios, to assess robustness, scalability, and generalizability.

Overall, RAPSO demonstrates significant improvements in the robotic milling of elastic materials and lays a solid foundation for future research in high-precision manufacturing applications.

## Figures and Tables

**Table 1:** Comparison of different groups.

No.	PSO (Pixel)	APSO (Pixel)	RAPSO (Pixel)	Improvement_1	Improvement_2
1	644	569	364	11.65%	36.03%
2	701	684	573	2.43%	16.23%
3	798	776	465	2.76%	40.08%
4	644	585	459	9.16%	21.54%

Improvement_1 refers to the improvement rate of APSO compared with PSO. Improvement_2 refers to the improvement rate of RAPSO compared with APSO.

## Data Availability

Data are contained within the article.
